# Effect of Cross-Orientation Normalization on Different Neural Measures in Macaque Primary Visual Cortex

**DOI:** 10.1093/texcom/tgab009

**Published:** 2021-02-10

**Authors:** Aritra Das, Supratim Ray

**Affiliations:** Centre for Neuroscience, Indian Institute of Science, Bangalore 560012, India

**Keywords:** area V1, contrast, gamma, normalization, SSVEP

## Abstract

Divisive normalization is a canonical mechanism that can explain a variety of sensory phenomena. While normalization models have been used to explain spiking activity in response to different stimulus/behavioral conditions in multiple brain areas, it is unclear whether similar models can also explain modulation in population-level neural measures such as power at various frequencies in local field potentials (LFPs) or steady-state visually evoked potential (SSVEP) that is produced by flickering stimuli and popular in electroencephalogram studies. To address this, we manipulated normalization strength by presenting static as well as flickering orthogonal superimposed gratings (plaids) at varying contrasts to 2 female monkeys while recording multiunit activity (MUA) and LFP from the primary visual cortex and quantified the modulation in MUA, gamma (32–80 Hz), high-gamma (104–248 Hz) power, as well as SSVEP. Even under similar stimulus conditions, normalization strength was different for the 4 measures and increased as: spikes, high-gamma, SSVEP, and gamma. However, these results could be explained using a normalization model that was modified for population responses, by varying the tuned normalization parameter and semisaturation constant. Our results show that different neural measures can reflect the effect of stimulus normalization in different ways, which can be modeled by a simple normalization model.

## Introduction

Divisive normalization refers to a canonical computation in which the response of a neuron is divided by the summed response of a larger (normalization) pool of neurons (Heeger 1992; Carandini and Heeger 1994; Carandini et al. 1997; Lee and Maunsell 2009; Reynolds and Heeger 2009; Ni et al. 2012, 2019). Normalization models have been used to explain a variety of sensory phenomena, such as saturation of the contrast response function (CRF; Heeger 1992; Carandini et al. 1997; Reynolds and Heeger 2009) and cross-orientation suppression (Busse et al. 2009; Brouwer and Heeger 2011). CRF saturation is a nonlinear phenomenon in which the response of a neuron increases linearly at lower contrasts but saturates at higher contrasts. Cross-orientation suppression is a phenomenon in which the response of a neuron to an optimally orientated grating stimulus is suppressed by a superimposed orthogonal grating, that is, plaid stimulus (Adelson and Movshon 1982; Freeman et al. 2002), even though the orthogonal grating produces little or no response when presented alone. These sensory phenomena have been well explained using a normalization model (Carandini and Heeger 2011). More recently, normalization has also been used to explain a variety of cognitive phenomena such as selective attention, predominantly to explain different ways in which selective attention can change the CRF (Lee and Maunsell 2009; Reynolds and Heeger 2009).

Cross-orientation suppression has also been studied in population signals such as the local field potential (LFP) and electroencephalogram (EEG), where the effect is often quite distinct from what is observed in spiking activity. For example, Lima et al. (2010) showed that gamma oscillations (30–80 Hz) induced by 2 orthogonal gratings when presented alone, get strongly suppressed when presented together as a plaid. Strength of gamma power in LFP was also shown to be dependent on normalization mechanisms (Ray et al. 2013). Likewise, the so-called steady-state visually evoked potential (SSVEP) that is produced by presenting a flickering visual stimulus at a particular frequency and is typically used in EEG recordings get suppressed in presence of another visual stimulus flickering at a different frequency (Burr and Morrone 1987; Ross and Speed 1991) in an asymmetric fashion, with low-frequency SSVEP tags causing more suppression than high-frequency tags (Salelkar and Ray 2020). Some of these interactions have also been explained based on normalization models (Candy et al. 2001; Busse et al. 2009; Tsai et al. 2012; Baker and Wade 2017; Cunningham et al. 2017). Recent studies have also looked at the effect of cross-orientation suppression on population responses such as visual evoked potentials (Busse et al. 2009)and noise correlations (Ruff and Cohen 2016; Ruff et al. 2016). However, typically, different studies have only modeled 1 neural measure (spiking activity, power in different bands, and SSVEP), and no study, to our knowledge, has modeled the suppression across various measures using a single normalization model. Such a comparison is crucial to understand the neural mechanisms underlying cognitive processes such as attention that are also thought to be linked to normalization. For example, single-unit studies have shown that selective attention can either lead to a multiplicative increase in firing rates (response gain; Williford and Maunsell 2006) or shift the CRF to the left (contrast gain; Reynolds et al. 2000; Martínez-Trujillo et al. 2002). While some SSVEP studies have also shown response gain (Joon Kim et al. 2007), to integrate these findings in a common framework, it is important to first test whether different measures are affected in similar ways due to normalization or attention.

To address this, we recorded spikes and LFP from the primary visual cortex (V1) of 2 passively fixating monkeys while presenting cross-oriented superimposed gratings (plaids) that were either static or counterphasing at 8 cycles per second. From the static and counterphasing gratings, we obtained spiking activity and SSVEP, respectively. In addition, from the LFP for the static condition, we focused on 2 features in the LFP: gamma oscillations, which are thought to index excitation–inhibition interactions (Atallah and Scanziani 2009), and high-gamma power (104-248 Hz), which are thought to reflect the overall firing activity of neurons near the microelectrode (Ray et al. 2008; Manning et al. 2009; Ray and Maunsell 2011). The band-powers and SSVEP power were measured from the respective band pass filtered data for static and counterphasing stimuli recorded by a microelectrode array. We then modified the normalization model for population responses and compared the parameters across these 4 neural measures.

## Materials and Methods

### Animal Recordings

All the animal experiments were performed in compliance with the guidelines approved by the Institutional Animal Ethics Committee of the Indian Institute of Science and the Committee for the Purpose of Control and Supervision of Experiments on Animals. Two adult female bonnet monkeys (*Macaca* radiata; Monkey 1: ~ 3.3 kg, 15 years old and Monkey 2: ~ 4 kg, 18years old) were used in this study. For each monkey, a titanium head post was implanted over the anterior/frontal region of the skull under general anesthesia. After recovery, the monkeys were trained for a visual passive fixation task. Once the monkeys were sufficiently trained to successfully perform the task, each of them was operated under general anesthesia and implanted with a custom-made hybrid electrode array in the macaque area V1 in the left cerebral hemisphere. The hybrid array had 81 (9 × 9) microelectrodes (Blackrock microsystems) and 9 (3 × 3) electro- corticogram (ECoG) electrodes (Ad-Tech Medical Instrument Corporation), both attached to the same connector made by Blackrock microsystems. This electrode arrangement was used for studies reported elsewhere (Dubey and Ray 2019,^2020^); however, for the present study, the data were only analyzed from 81 microelectrodes. Each of microelectrodes were 1 mm long, separated by 400 μm with a tip diameter of 3-5 μm. Area V1 was identified with stereotactic coordinates and by visual inspection of the lunate and the superior temporal sulci. The microelectrode array was placed 10–15 mm from the occipital ridge and 10–15 mm lateral from the midline. The entire length of the microelectrode penetrated the cortex. The reference wires were placed over the dura near the edge of the craniotomy or secured to the metal strap used to secure the bone on the craniotomy. The receptive fields of the neurons recorded from the microelectrodes were centered in the lower right quadrant of the visual field at an eccentricity of ~ 3.5° to ~ 4.5° in Monkey 1 and ~1.6° to ~ 1.8° in Monkey 2.

The mean impedance of microelectrodes was ~ 0.6 MΩ (range 0.1-1.8 MΩ) at 1 kHz for both the monkeys. Both LFP and multiunit activity (MUA) were recorded using the Cerebus Neural Signal Processor (Blackrock Microsystems). LFP was obtained by bandpass filtering the raw data between 0.3 Hz (Butterworth filter, first order, analog) and 500 Hz (Butterworth filter, fourth order, digital) sampled at 2 kHz and digitized at 16-bit resolution. MUA was obtained by filtering the raw signal between 250 Hz (Butterworth filter, fourth order, digital) and 7500 Hz (Butterworth filter, third order, analog), followed by an amplitude threshold (set at ~ 5 standard deviations [SDs]) of the signal for both the monkeys. The MUA, which we also refer to as a “neuron,” was based on this amplitude threshold crossing and was not sorted further.

### Behavioral Task

For the behavioral task, the monkeys were placed inside a monkey chair with their head fixed by the head post mounted on the chair and grating or plaid stimuli of varying contrasts and orientations were displayed on a monitor (BenQ XL2411 LCD, at 1280 × 720 resolution, 100 Hz refresh rate, gamma-corrected and calibrated to a mean luminance of 60 cd/m^2^ on the monitor surface using i1Display Pro; x-rite PANTONE) placed ~ 50 cm from their eyes. The monkeys and the display setup were placed inside a Faraday enclosure lined with copper with a dedicated grounding separate from the main power supply to provide isolation from external electrical noise.

Each monkey performed a passive fixation task in which fixation had to be maintained at a small white dot (0.10° radius) at the center of the screen for the duration of a trial, which varied between 2.5 and 3.5 s. Each trial began with fixation, followed by an initial blank gray screen of 1000 ms; presented 2–3 stimuli for 500 ms each, with an interstimulus period of 500 ms. The monkeys were rewarded with a drop of juice for successfully maintaining fixation within 2° of the fixation spot during the entire duration of the trial. The stimuli were achromatic plaid stimulus of radius 1°, composed of 2 gratings with orthogonal orientations at a spatial frequency of 4 cycles per degree (cpd).

The stimuli spanned only the receptive fields of the microelectrodes but not of the ECoG electrodes. The contrast of each of the component grating during any stimulus presentation could take 5 possible values: 0%, 6.2%, 12.5%, 25%, and 50%, whereas the temporal frequency of each of the component grating during any stimulus presentation could take 2 possible values: 0 Hz (static grating) or 8 Hz (counterphasing grating). The orthogonal pair of orientations chosen for different sessions were 0–90, 22.5–112.5, 45–135, and 67.5–157.5°.

Data from 2 monkeys were collected in 13 and 9 recording sessions, respectively. Only correct trials (in which the monkeys maintained fixation throughout the duration of the trials) were used for analysis. We only analyzed the plaid stimuli data when both the component gratings were either static or counterphasing at 8 Hz. On average, we obtained 33 ± 1 stimulus repeats for each of the stimuli conditions.

### Electrodes Selection

We chose electrodes for which the following 3 conditions were met: 1) at least 15 spikes across all trials were recorded during stimulus period of 150–400 ms for either of the grating stimulus at 50% contrast, 2) signal-to-noise (SNR) ratio of the spike waveform (Kelly et al. 2007) was at least 2, and 3) receptive field centers were within 0.75° of the stimulus center in each of the recording sessions. This yielded 143 electrodes (37 unique) for Monkey 1 and 48 electrodes (13 unique) for Monkey 2. For a proper comparison between spike and LFP measures, the same set of electrodes was also used for LFP and SSVEP analysis. To account for potential differences in signal variability of MUA versus LFP across sessions, for each unique electrode, we averaged data across sessions, yielding 50 unique electrodes from both monkeys combined. This set was subsequently used for all 4 neural measures. The results were similar when analysis was performed without combining data for each electrode across sessions (Supplementary Fig. 3).

### Data Analysis

All data were analyzed using custom codes written in MATLAB (The MathWorks, RRID: SCR_001622). Individual data analysis methods are briefly summarized below.

### Absolute and Relative Power Spectral Density Plots

Power spectral densities (PSDs) for different stimulus conditions were computed using multitaper method with 1 taper using the Chronux toolbox (Bokil et al. 2010; http://chronux.org/, RRID: SCR_005547; Figs 3, 5, and 6). The analysis period was selected between 150 and 400 ms after stimulus onset to avoid stimulus onset-related transients and compared against a “baseline period” between -300 and -50 ms of stimulus onset (a gray screen). Absolute PSDs were expressed in logarithmic units (base 10), and change in PSDs was plotted with respect to the baseline response (common baseline for all plaid contrast combinations) for each plaid contrast condition:

$$\Delta {\text{PSD}}_{i}=10\left(\log_{10}{\left(\text{ST}\right)}_{i}-{\text{BL}}_{\text{common}}\right)$$

Here i represents a contrast condition of a plaid; Δ PSD_i_ represents the change in PSD in decibels; (ST)_i_ denotes the PSD in the stimulus epoch for stimulus condition i; and BL_common_ denotes the mean baseline PSD across all 25 (5 × 5) contrast conditions, that is, BL_common_ = mean [log_10_ (BL_i_)]. Absolute PSDs and change in PSDs in Figure 5A were computed for static stimuli, and whereas the same in Figure 6A were computed for counterphasing stimuli.

For the changes in gamma power and high-gamma power as shown in Figure 5 for static stimuli, we first averaged the raw power between 32–80 and 104–248 Hz for both stimulus and baseline periods. For the changes in SSVEP power as shown in Figure 6 for counterphasing stimuli, we computed the raw power at 16 Hz (twice the counterphasing frequency, i.e., 8 Hz) for both stimulus and baseline periods. We then repeated the same procedure as described above to compute the change in power.

### Time–Frequency Analysis

We computed the time–frequency power spectra using multitaper method with 1 taper (Fig. 3). We used a moving window of size 100 ms and step size of 25 ms, giving a frequency resolution of 10 Hz. We computed baseline power by averaging power across baseline period in logarithmic units (base 10) for each frequency. We further computed the common baseline time–frequency power spectra by averaging this baseline power across all 25 contrast conditions. We computed the changes in time–frequency power spectra at each of the contrast conditions by subtracting the common baseline power from its respective time–frequency power spectra.

### Normalization Model

The standard normalization model is as follows:

(1) $$R_{1,2}=\frac{c_{1}L_{1}+c_{2}L_{2}}{c_{1}+c_{2}+\sigma }$$

where R_1,2_ is the mean response, c_1_ and c_2_ are the contrasts of the 2gratings; L_1_ and L_2_ are the responses of the site’s linear receptive field to the individual gratings at unit contrast respectively; *σ* is a positive-term that denotes the semisaturation constant for the CRF of an electrode. This equation is similar to the normalization equations described in previous studies (Heeger 1992; Carandini et al. 1997). The linear responses to the plaid (c_1_L_1_ + c_2_L_2_) is “divisively normalized” by the overall activity of the normalization pool, which is proportional to the contrasts of the 2 gratings, c_1_ and c_2_. We refer to this model as the “untuned” normalization model because the strength of the normalization signal is not dependent on the orientation of the gratings.

Recently, the standard normalization model has been modified to include a tuned normalization component (Ni et al. 2012; Ni and Maunsell 2017). In this case, the normalization model is as follows:

(2) $$R_{1,2}=\frac{c_{1}L_{1}+c_{2}L_{2}}{c_{1}+\alpha c_{2}+\sigma }$$

Here, a tuned normalization parameter (α) is introduced to the standard divisive normalization model (equation 1) in the denominator to describe the extent of normalization contributed by one population on the other. Normalization is “untuned” for *α* = 1 and becomes “tuned” as *α* deviates away from 1.

Here we describe a normalization model for population signals such as LFP and EEG. Note that the normalization models mentioned above describe the activity of an individual neuron. Neurons in V1 are highly orientation selective, so a population of neurons that prefer the first stimulus will hardly respond to the second stimulus, and their response can be approximated (by setting L_2_ to zero) as: $\frac{c_{1}L_{1}}{c_{1}+\alpha c_{2}+\sigma }$ Similarly, a population of neurons that prefer the second stimulus can be approximated as $\frac{c_{2}L_{2}}{\alpha c_{1}+c_{2}+\sigma }$. The population signal, which is the sum of the activity of both these subpopulations, can therefore be approximated as:

(3) $$R_{1,2}=\frac{c_{1}L_{1}}{c_{1}+\alpha c_{2}+\sigma }+\frac{c_{2}L_{2}}{\alpha c_{1}+c_{2}+\sigma }$$

Interestingly, this normalization model is similar to the “equal-maximum-suppression stimulus-tuned” normalization model proposed by Ni and colleagues for fitting normalization responses of neuronal spike activity in middle-temporal (MT) cortex when the relative locations of nonoverlapping stimuli are varied within the receptive field of MT neurons (Ni and Maunsell 2017). In their case as well, this model outperformed the other normalization models.

Although previous studies have used exponents to scale responses in CRFs with (Reynolds and Heeger 2009; Itthipuripat et al. 2014) or without attention (Tsai et al. 2012) using normalization models, we have avoided it here for 2 reasons. First, this reduces one free parameter. Second, we wanted our model to be consistent with the model proposed by Ni and Maunsell (2017).

### Model Fitting

The fitting procedure was the same for each of the neural measures. For a given neural measure, R1,2 is the actual neural response for a given contrast condition obtained from the experiments. The free parameters in the standard (untuned) normalization model were L_1_, L_2_, and *σ*, whereas the free parameters in the tuned and population normalization models were L_1_, L_2_, *a,* and σ. The starting points for each of the free parameters were set separately. L_1_ was initialized by setting c_2_ = 0 (contrast of grating 2 is 0%); L_2_ was initialized by setting c_1_ = 0 (contrast of grating 1 is 0%), and alpha was initialized by setting c_1_ = c_2_ = 0.5 (contrast of grating 1 and 2 are 50% each). Sigma was initialized at 0.1 and constrained between 0 and 5. We fit the free parameters of the relevant model(s) to the 25 combinations of contrasts using the “fminsearch” function in MATLAB. The predicted responses were then obtained for each of the 25 contrast conditions by using the estimated parameters to respective normalization equation. The sum of squared estimate of errors (SSED) was calculated between the actual and predicted response for all 25 contrast conditions. The variability in the actual data was calculated by the sum of squared estimate of errors around the mean (SSE_M_) for 25 contrast conditions. The ratio (SSE_D_/SSE_M_) was the fraction of data that could not be explained by the model and thus explained variance was calculated by subtracting the ratio from 1 and converted to percentage. We present fit results for the entire population by fitting the median response across all electrodes using population normalization model (Figs 4D, 5D, and 6D). We also present fit results for individual electrodes by using each of the normalization models (Fig. 7A).

Because the different normalization models had different number of free parameters, to compare their performance, we calculated the corrected Akaike information criteria (AIC_c_)values for each of the electrodes for each of the 3 models (Hurvich and Tsai 1989; Motulsky and Christopoulos 2004):${\text{AIC}}_{\text{c}}=\text{N}\times \log_{e}\frac{{\text{SSE}}_{\text{D}}}{\text{N}}+2\text{K}+\frac{2\text{K}(\text{K}+1)}{\text{N}-\text{K}-1}$ where SSE_D_ is the sum of squared estimate of errors as before, N is the number of observations, and K is the number of fitted free parameters. The number of observations for each model was 25 for the number of different contrast conditions. There were 3 free parameters for our “untuned” model and 4 free parameters each for “tuned” and “population” models. We also included the SSE_D_ itself as another degree of freedom (Motulsky and Christopoulos 2004), resulting in fitted parameters to increase to 4 for “untuned” model and 5 each for “tuned” and “population” model. According to this formula, the absolute value of the AIC_c_ has no meaning since it only depends on the absolute values of sum of squared error (SSE_D_ in the formula). In particular, the AIC_c_ values can also be negative. The important comparison is how this value changes across models. The absolute difference between AIC_c_ values is important in selection of the preferred model and not the absolute AIC_c_ values (Motulsky and Christopoulos 2004).

### Statistical Analysis

Unless noted otherwise, all the statistical analyses were computed for the sample size of N = 50 unique electrodes obtained from combined dataset of 2 monkeys. The standard error of medians (SEM) of normalization index (N.I.) (a suppression index described in Results section), normalization parameter (α), semisaturation constant (σ), explained variance and AIC_c_ values of normalization models were computed using bootstrapping method. In this method, from a sample of 50 elements, a new sample of 50 elements was chosen randomly with replacement to the original sample (termed as bootstrap sample). The median of this bootstrap sample was computed. This process was iterated 1000 times to generate 1000 medians. The SD of this population of medians was reported as SEM in Figure 7A–D.

Statistical tests were performed between different neural measures to test whether their distributions were significantly different from each other. The null hypotheses of these tests assumed that the medians of the sample distribution between 2 neural measures were from the same distribution. Nonparametric tests (Kruskal–Wallis test) were performed between different neural measures in pairs for their sample distributions of N.I., tuned normalization parameter (α), and semisaturation constant (σ).

## Results

We analyzed the effect of stimulus normalization on 4 different neural measures (spikes, gamma power, high-gamma power, and SSVEP) by recording spikes and LFP from the primary visual cortex (area V1) of 2 passively fixating monkeys while presenting static (for spikes, gamma, and high-gamma power) or counterphasing (for SSVEP) plaids. Results were similar for the 2 monkeys and therefore the data were pooled, yielding a total of 191 electrodes across 22 recording sessions. We further combined data from individual electrodes across sessions, yielding 50 unique electrodes (see Materials and Methods for details).

**Figure 1:**
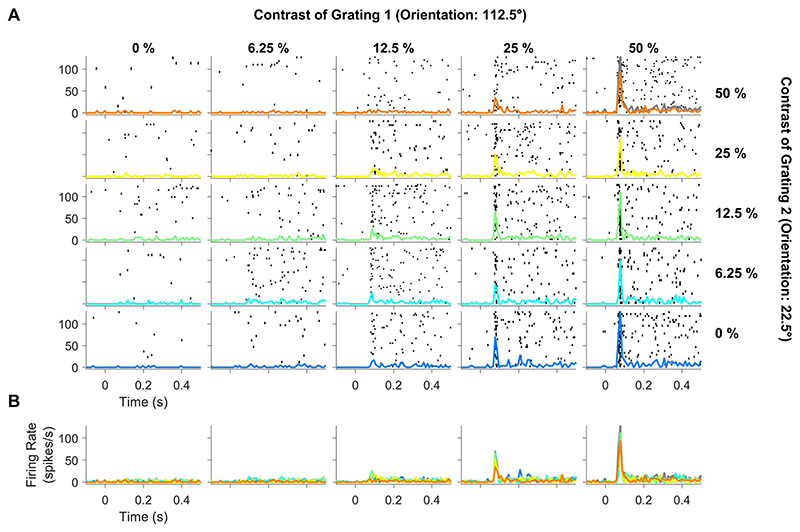
Spiking activity profile for different contrast conditions of the plaid stimuli for a representative electrode, which preferred one component grating (112.5°) but not the other (22.5°). (A) Raster plots showing spiking activity in individual trials for each contrast combination during presentation of static orthogonal plaid stimuli (0–500 ms). The columns (left to right) represent increase in contrast of the component grating 1 (from 0% to 50% in steps of multiplicative factor of 2) and rows (bottom to top) indicate increase in contrast of the component grating 2 in the same manner as component grating 1. Trial-averaged PSTH is overlaid on individual raster plots (blue to orange traces highlight increase in contrast for component grating 2). Gray trace in the top-right tile shows the sum of responses to individual component gratings (top-left and bottom-right traces). (B) PSTH plots of varying contrast conditions of component grating 2 is overlaid together (all 5 rows collapsed into a single row).

Figure 1A shows the raster plots and peristimulus time histogram (PSTH) of one example electrode from a single recording session when 2 overlapping static gratings of 112.5° (preferred) and 22.5° (null) orientations were presented at 5 contrast levels each (25 contrast conditions). Although the null orientation by itself produced negligible response at any contrast level (first column), it nonetheless suppressed the response due to the preferred stimulus, which can be observed by comparing the last row that corresponds to the preferred only condition to the first row, which corresponds to the same preferred stimulus with a superimposed null stimulus at 50% contrast. To highlight the suppression, we also computed the sum of responses to individual gratings at 50% contrast (sum of the traces in top-left and bottom-right tiles, indicated by a gray line in the top-right tile) and overlaid on the plaid response at 50% contrast (orange line in the top-right tile). Figure 1B shows the overlaid PSTHs for different contrast values of the null grating (blue to orange traces indicate increase in contrast) for each value of the preferred contrast level, which shows suppression of the initial transient response as the contrast of the null stimulus increases.

**Figure 2:**
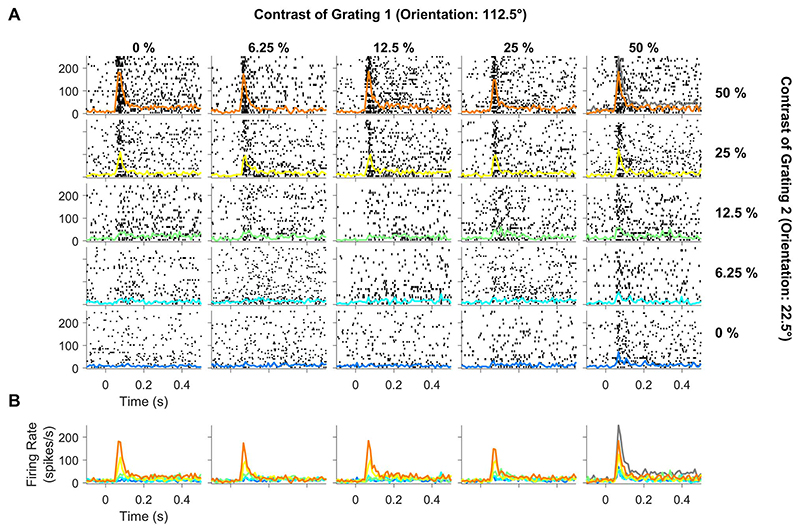
Spiking activity profile for different contrast conditions of the plaid stimuli for a representative electrode that was responsive to both component gratings. Same layout as Figure 1.

Because a single orthogonal pair of orientations was used for each session, they were not the preferred and null orientations for most neurons. Instead, most neurons responded to both gratings. Figure 2A shows an example of a site where both gratings of the plaid produced a significant response (following the same layout in Fig. 1A). Cross-orientation suppression was not evident directly but could be observed by comparing the responses to the plaid at 50% contrast (orange trace in the top-right tile) with the sum of responses to individual gratings at 50% contrast (gray trace in the top-right tile).

**Figure 3:**
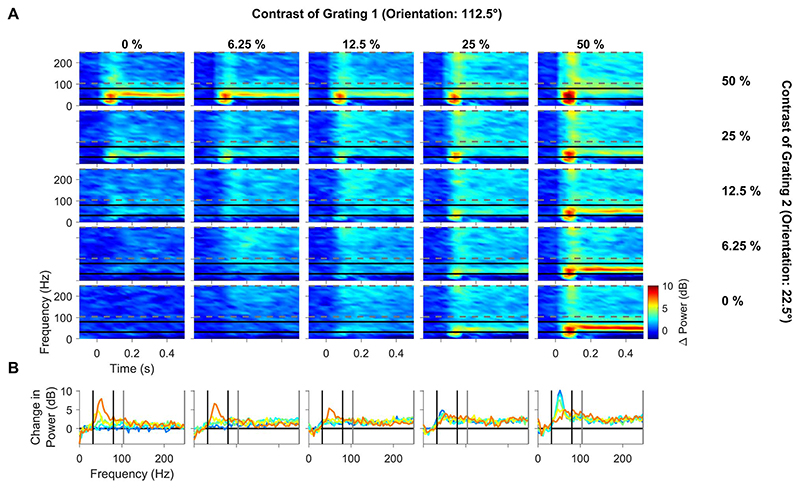
Difference in time–frequency energy profile and PSD profile. (A) Time–frequency energy difference plots for the same example electrode as in Figure 1 (in dB) showing the difference in energy from baseline energy (-300 to -50 ms, 0 denotes the stimulus onset, difference computed separately for each frequency) for contrast combinations of the static plaid stimuli. Gamma and high-gamma range is denoted by solid black horizontal lines (32–80 Hz) and gray horizontal dashed lines (104– 248 Hz). (B) Change in PSD of all contrast combinations of component grating 2 overlaid together (same arrangement to Fig. 1); gamma and high-gamma ranges are denoted by solid black vertical lines (32–80 Hz) and gray vertical lines (104–248 Hz).

Figure 3A shows the change in power with respect to a baseline period (-300 to -50 ms before stimulus onset; see Materials and Methods) of the LFP recorded from the same example electrode as Figure 1. While gratings at either of the 2 orientations at 25% and 50% contrast produced strong gamma oscillations (top-left and bottom-right panels), these oscillations were severely attenuated for plaid stimuli (top-right panel). This effect has been previously reported in macaque V1 area by Lima et al. 2010. Figure 3B shows the change in PSD computed during 150–400 ms after stimulus onset with respect to the baseline period, following the same format as in Figure 1B, to clearly visualize the change in power for each contrast condition and the effect of normalization at different frequencies.

High-gamma power (104–248 Hz) increased after stimulus onset, but the increase was less for plaids compared with gratings, best observed at mid-level contrasts (middle panels in Fig. 3B) for this electrode. Similar to firing rates, the effect was more subtle than what was observed at gamma frequencies. We also observed a strong suppression at alpha frequencies (8– 12 Hz). However, the effect was difficult to quantify because the stimuli were presented only for 500 ms and analyzed over only 250 ms, yielding a frequency resolution of 4 Hz and consequently only 2 points in the alpha range, which was insufficient to detect potential shifts in alpha peak frequencies.

Further, alpha suppression started relatively later for plaid stimuli (roughly 300 ms) compared with the grating conditions (roughly 200 ms; compare alpha ranges in top vs. bottom- rightmost plot in Fig. 3A). Therefore, power in the alpha range was not analyzed further.

### Effect of Stimulus Normalization on Population Spiking Activity in Area V1

**Figure 4:**
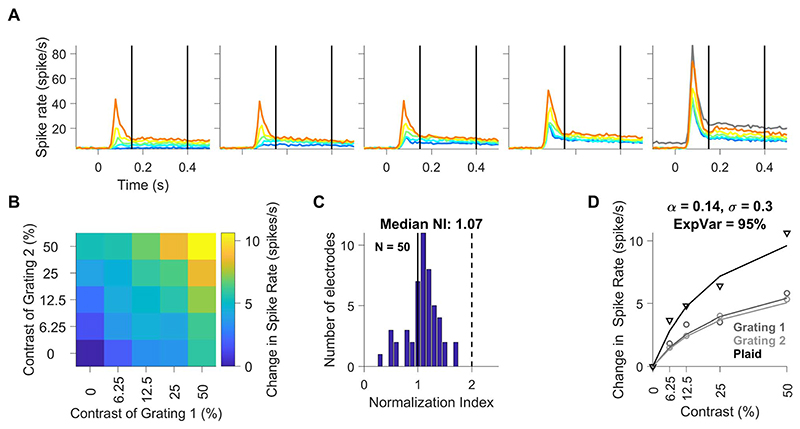
Population spiking activity profile. (A) Mean firing rate for all contrast combinations of plaid stimuli across all 50 unique electrodes during time period of -100 to 500 ms with respect to stimulus onset. The stimulus period of 150-400 ms is denoted by thick vertical lines. Error bars have been omitted for clarity. The plots (left to right) in panel A represent increasing contrast of grating 1, whereas individual traces (blue to orange) in each of the plots indicate increasing contrast of grating 2. Gray trace in the rightmost plot shows the sum of responses to individual component gratings at 50% contrast. (B) Median change in population spike response of all V1 units to static plaid stimuli as a function of the contrast of each component grating (x-axes correspond to component grating 1 and y-axes correspond to component grating 2). (C) Histogram of N.I. for spike responses of all unique units recorded in V1; vertical black solid-dashed lines indicate the boundary range of units with N.I. values between 1 and 2, respectively. (D) Median population responses for grating 1 (dark gray circles), grating 2 (light gray circles) and plaid (black triangles) as a function of contrast. The lines show fit of the median population responses by a single population normalization model (same color scheme as markers); caption indicates values of fitted parameters and explained variance for the mean population spiking activity.

Figure 4A shows the mean firing rates of 50 unique electrodes from 2 monkeys, in the same format as Figure 1B. Here, we simply averaged the responses across electrodes irrespective of their individual orientation preference. Figure 4B shows the median change in spike rates during 150–400 ms after stimulus onset relative to baseline, as a function of the contrast of each grating (x- and y-axes for component gratings 1 and 2, respectively). To quantify the suppression, we calculated N.I. for each electrode (as proposed by Ruff and Cohen, 2016) as the ratio of the sum of the responses to individual gratings at 50% contrast to the response to their superposition (gray and orange traces in Fig. 4A). According to this equation, N.I. value of 2 indicate perfect normalization, where the response for plaid stimulus at 50% contrast is the average of both the grating stimuli at 50% contrast, whereas value of 1 indicates no normalization (plaid response is the sum of individual grating response). Values >2 show very strong normalization where the response for plaid stimulus at 50% contrast is even lower than average of both the grating stimuli at 50% contrast.

Figure 4C shows the histogram of N.I. values for the population. The median N.I. ± SEM (computed using bootstrap method) of all multiunits was 1.07 ± 0.03, indicating mild cross-orientation suppression in our multiunit data. These N.I. values are slightly lower than the values obtained for multiunits by Ruff and colleagues for MUA in V1 (1.14). The weaker normalization could be due to the use of a fixed spatial frequency, which was optimized to produce strong gamma oscillations. However, this was not due to the use of a suboptimal pair of orientations. In control analyses, we found no correlation between the N.I. values and difference between the preferred and stimulus orientations for a neuron, suggesting that normalization is largely a property of a particular neuron rather than the response to specific stimuli, as shown previously by Ruff and Cohen (2016). The lower N.I. values could also be due to the use of static stimuli, which had lower sustained firing rates in the analysis period. In comparison, N.I. value for spike responses for counterphasing plaids was 1.22 ± 0.04 (Supplementary Fig. 1).

A normalization model, modified for population responses (equation 3), provided good fits to the MUA data as well. Figure 4D shows the change in median spike rates of the population for 2 gratings at 5 contrast levels (circles in 2 shades of gray), their superposition to form a plaid (black triangles), as well as the fit of the normalization model (corresponding traces in gray and black; all 25 contrast levels were used to fit the model). This model explained 95% of the variability in this dataset. The performance of the model when applied to individual electrodes is described later (Fig. 7).

### Effect of Stimulus Normalization on Gamma and High-Gamma Power in Area V1

**Figure 5:**
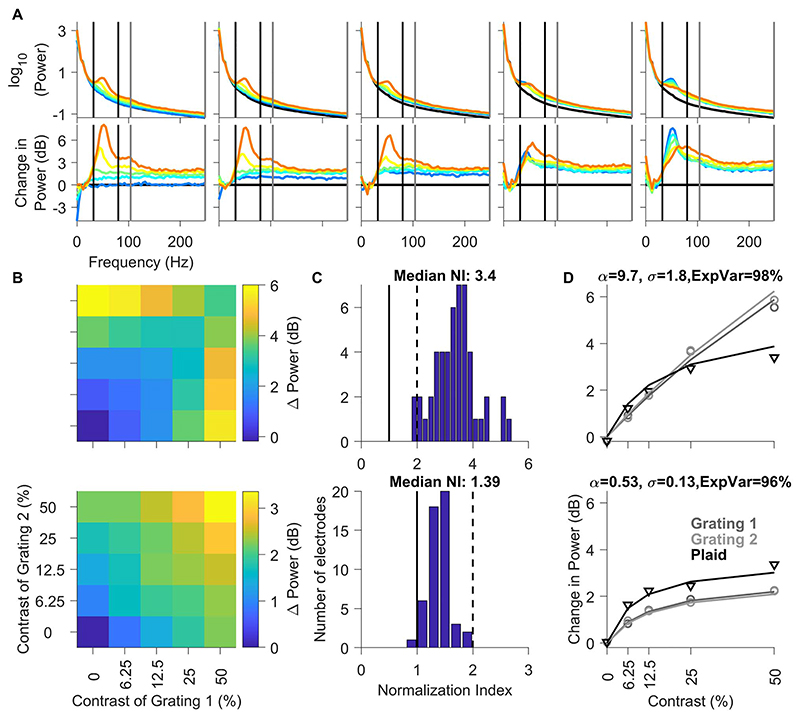
Changes in gamma and high-gamma power profile as a function of contrasts of component gratings. Solid black and solid gray vertical lines indicate gamma and high-gamma range, respectively. (A) Top row: PSDs during stimulus period (150–400 ms after stimulus onset) as a function of contrast of component gratings of the static plaid stimuli, layout same as in preceding figures, black trace indicates the mean baseline for all contrast combinations. Bottom row: Corresponding change in PSDs during stimulus period (150–400 ms after stimulus onset) with respect to prestimulus baseline (-300 to -50 ms) as a function of contrast of component gratings of the static plaid stimuli. (B) Median change in population response of all electrodes to static plaid stimuli as a function of the contrast of each component grating (x-axes correspond to component grating 1 and y-axes correspond to component grating 2). (C) Histogram of N.I. of median population responses all unique electrodes; vertical black solid-dashed lines indicate the range of units with N.I. values between 1 and 2. (D) Median population responses for grating 1 (dark gray circles), grating 2 (light gray circles), and plaid (black triangles) as a function of contrast. The lines show fit of the median population responses by a single population normalization model for each of the conditions (same color scheme); caption indicates values of fitted parameters and explained variance for the mean population spiking activity. (B–D) Change in gamma power (top); high-gamma power (bottom).

Next, we analyzed the effect of stimulus normalization on LFP gamma band power and high-gamma band power averaged across all unique electrodes. The top panel in Figure 5A shows the PSDs of different contrast conditions of the plaid stimuli for the interval between 150 and 400 ms. The 5 plots in the top panel (left to right) denote increasing contrasts of grating 1 and individual blue to orange traces represent increasing contrasts of grating 2. Black trace denotes the mean PSD across all contrast conditions for baseline period of -300 to -50 ms with respect to stimulus onset. The lower plots show the change in power during stimulus period from this baseline, similar to the scheme presented in Figure 3B. Similar to the results shown in the example electrode in Figure 3, we observed increases in gamma band power and peak frequency with increasing contrast of the grating 2 (leftmost plot in top panel). We also observed a drastic reduction in gamma power as well as an increase in peak frequency when gratings were superimposed to form a plaid (best observed in the rightmost panel). The leftmost panel in Figure 5A also shows that gamma oscillations become faster with increasing contrast of the gratings, as shown previously (Ray and Maunsell 2010), whereas the rightmost panel shows that plaids have less gamma power but a higher center frequency than gratings, as shown by Lima et al. (2010).

As before, we quantified these results by computing the total change in power in the gamma and high-gamma bands relative to the baseline power in these bands (top and bottom rows of Fig. 5B) and calculated the N.I. for change in gamma and high-gamma power in the same manner as spikes (Fig. 5C). N.I for gamma varied between 1.8 and 6 (median: 3.4 ± 0.12), with majority of electrodes showing suprastrong cross-orientation suppression (N.I. >2). For high-gamma power, the median N.I. was 1.39 ± 0.03, significantly higher than the N.I. for spikes (Kruskal–Wallis test, P = 1.15× 10^-9^). Importantly, the same normalization model as used above could explain the variation in power in both bands with high accuracy (explained variance of 98% and 96% for gamma and high-gamma power of the population data; Fig. 5D) by taking different values of the tuned normalization parameter (alpha) and the semisaturation constant (sigma).

### Effect of Stimulus Normalization on SSVEP Power in Area V1

**Figure 6:**
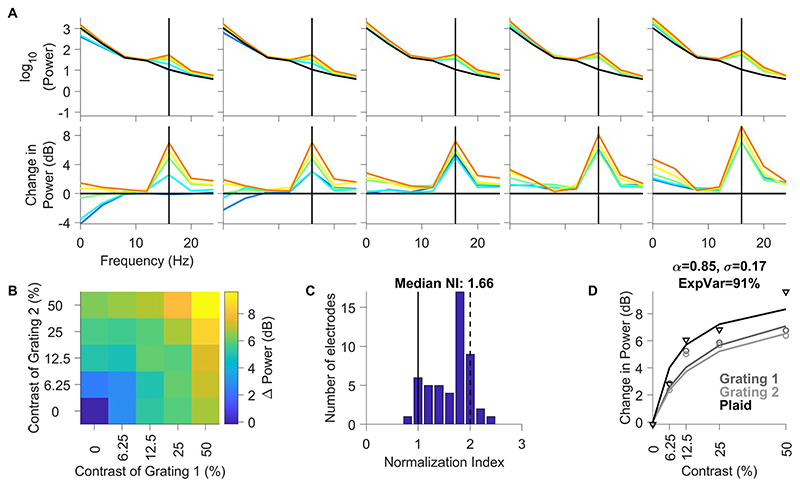
Changes in SSVEP power profile as a function of contrasts of component gratings of flickering Plaid. (A–D) Same layout as in Figure 5A–D. The black vertical lines in each of the plots in Panel A indicate the SSVEP power at 16 Hz (second harmonic of the counterphasing stimuli flickering at 8 Hz).

We then performed similar analysis for SSVEP power obtained from counterphasing stimuli, for which the strongest response was observed at twice the temporal frequency (16 Hz; Fig. 6A; same format as Fig. 5). Normalization in SSVEPs (median N.I.: 1.66 ± 0.08; Fig. 6C) was stronger than high-gamma power (Kruskal–Wallis test, P = 7.03 × 10^-3^), although significantly weaker than gamma (Kruskal–Wallis test, P = 1.79× 10^-17^). The normalization model used earlier provided excellent fit to this dataset as well (explained variance of 91%; Fig. 6D). Comparison of the fitted parameters on median of the population data (Figs 4D, 5D, and 6D) revealed that while the semisaturation constant (sigma) was similar for spikes, high-gamma and SSVEP (0.3, 0.13, and 0.17, respectively), the parameter alpha increased considerably (0.14, 0.53, and 0.85, respectively) to explain the increase in N.I. for these 3 measures. For gamma power, both sigma and alpha (1.8 and 9.7, respectively) were almost an order of magnitude larger, which explained the linear increase in gamma power with increasing contrast of component gratings and the strong suppression with superimposed gratings, respectively. We quantified this by comparing the model fits to individual electrodes below.

### Comparison of Effect of Stimulus Normalization on Different Neural Measures

Finally, we fitted the normalization model to individual electrodes to compare the fit quality and fitted parameters of all the 4 neural measures (spike rate, gamma power, high-gamma power, and SSVEP power).

**Figure 7:**
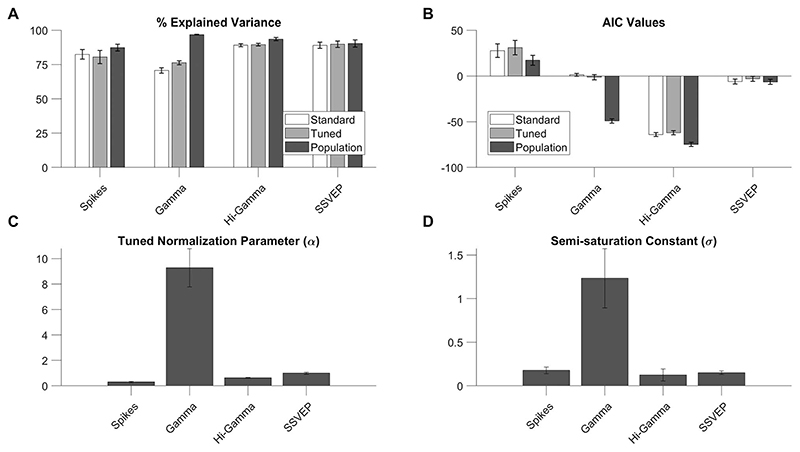
Comparison of normalization model parameters for all neural measures for 50 unique electrodes. (A) Bar plots (white to gray) showing median explained variance for the respective neural measures for the untuned, tuned, and population normalization models. (B) Bars (white to gray) represent median corrected AIC values for standard, tuned, and population normalization models (C) Bar plots of median normalization parameter (α) for all 4 neural measures for our population model. (D) Bar plots of median semisaturation constant (σ) for all 4 neural measures for our population model. Error bars indicate bootstrapped SEM.

First, we compared 3 normalization models—standard normalization model (equation 1), tuned normalization model (equation 2), and finally the population normalization model proposed here (equation 3). While the fit quality with this new “population normalization model” was high for all measures (87 ± 3%, 97 ± 0.2%, 94 ± 1%, and 90 ± 2% for spikes, gamma, high gamma, and SSVEP, respectively), other 2 models fared poorly, especially for gamma (Fig. 7A). To account for differences in free parameters (3, 4, and 4 for the 3 models), we compared the corrected AIC values for each electrode for each of 3 models (Fig. 7B). The median AIC values were not significantly different between the standard and tuned models (Kruskal-Wallis test, P = 0.41, 0.54, 0.55, and 0.50 for spikes, gamma, high-gamma, and SSVEP, respectively). However, the difference in corrected AIC values between population and tuned models was highly significant for gamma (Kruskal-Wallis test, P = 0.30, 7.33 × 10^-17^, 0.02, and 0.52 for spikes, gamma, high-gamma, and SSVEPs, respectively). We discuss the reasons behind the failure of the standard and tuned model for gamma power in the Discussion section. For the remaining section, we used the fits for the new population normalization model only.

The tuned normalization parameter *α* for the 4 measures were 0.3 ± 0.04, 9.28 ± 1.43, 0.63 ± 0.03, and 1 ± 0.09 (Fig. 7C). This parameter was significantly higher for gamma power than firing rate (P = 3.23 × 10^-17^, Kruskal-Wallis test), high-gamma power (P = 6.86 × 10^-18^, Kruskal-Wallis test), and SSVEP (P = 6.86 × 10^-18^, Kruskal-Wallis test). There was also significant difference between firing rate and high-gamma power (P = 4.19 × 10^-7^,į Kruskal-Wallis test), between firing rate and SSVEP power (P = 1.84× 10^-9^, Kruskal-Wallis test) and that between high- gamma power and SSVEP power (P = 5.59 × 10^-7^, Kruskal-Wallis test).

The median semisaturation constants (σ), which specifies how rapidly the CRF reaches semisaturation, were 0.18 ± 0.04, 1.23 ± 0.34, 0.12 ± 0.07, and 0.15 ± 0.02 for the 4 neural measures (Fig. 7D). While the values for gamma differed significantly from firing rate (P = 1.31× 10^-7^, Kruskal-Wallis test), high- gamma power (P = 1.64 × 10^-14^, Kruskal-Wallis test), and SSVEP (P = 5.04 × 10^-16^, Kruskal-Wallis test), none of the differences between firing rate and high-gamma power (P = 0.15, Kruskal- Wallis test), firing rate and SSVEP power (P = 0.11, Kruskal- Wallis test), and high-gamma power and SSVEP power (P = 0.53, Kruskal-Wallis test) were significant. Supplementary Figure 2 shows the same results separately for the 2 monkeys, along with data from individual electrodes. While there were minor differences across monkeys (potentially due to a smaller sample size for Monkey 2), the main results—superior performance of the population normalization model over the standard and tuned models for gamma and significantly larger normalization parameter and semisaturation constant for gamma compared to other measures—were consistent across monkeys. These results remained consistent even when data across individual sessions for a particular electrode were not pooled, which yielded 143 nonunique electrodes from Monkey 1 and 48 nonunique electrodes from Monkey 2 (Supplementary Fig. 3). Finally, results remained consistent when the analysis was restricted to the early stimulus period between 0 and 250 ms after stimulus onset (Supplementary Fig. 4). Although the gamma rhythm was less prominent in the first 250 ms, leading to lower median N.I. values (1.89 as compared with 3.37 for the late period as described earlier) and tuned normalization parameter (5.01 as compared with 9.28), our population normalization model was still able to outperform the other 2 models in explaining the change in gamma power (Kruskal–Wallis test on corrected AIC values, P = 0.20 between the standard and the tuned model and P = 2.87 × 10^-14^ between the tuned and the population model). The median semisaturation constant values became higher and more variable for all measures because transient responses were stronger and more variable across electrodes.

To test whether normalization strengths of different measures were correlated, we analyzed the Spearman correlation coefficient for N.I. values of different neural measures across the 50 unique electrodes. We found significant correlation in the N.I. values of MUA and high-gamma (Spearman correlation coefficient, r = 0.39, P = 0.02 in Monkey 1, r = 0.75, P = 4.4 × 10^-3^ for Monkey 2), which is not unexpected because high-gamma is thought to reflect population firing rate (Ray and Maunsell 2011). Correlations between other measures were not consistent/signif- icant across monkeys.

### Eye Position Analysis

Because small stimuli were placed on receptive fields that were close to the boundary of fixation window (specially for Monkey 2), we compared the horizontal and vertical eye positions for the 25 different contrast conditions, for both static and counterphasing stimuli. We performed a grand mean for horizontal eye-position, vertical eye-position (mean over all time points between -500 to 500 ms and subsequently for all stimuli repeats) for each of the 25 contrast conditions. We did not find any significant difference in horizontal and vertical eye positions across 25 different contrast conditions for either static or counterphase stimuli in either monkey (1-way analysis of variance [ANOVA] test: Eye-Position Horizontal, Monkey 1: P_static_ = 0.11, P_counterphase_ = 0.66, Monkey 2: P_static_ = 0.31, P_counterphase_ = 0.76, Eye-Position Vertical, Monkey 1: P_static_ = 0.17, P_counterphase_ = 0.81, Monkey 2: P_static_ = 0.45, P_counterphase_ = 0.22). We also detected microsaccades using a threshold-based method described earlier (Murty et al. 2018, 2020), initially proposed by Engbert (2006). Specifically, we identified microsaccades as eye movements with velocities that crossed a specified threshold for at least a specified duration of time. We set the velocity threshold between 3 and 6 times the SD of eye-velocities and minimum microsaccade duration between 10 and 15 ms for an individual monkey to maximize the correlation between peak velocity and amplitude of all microsaccades for that monkey (also called a “main sequence,” see Engbert 2006 for details), while maintaining the minimum microsaccade velocity at 10°/s and the microsaccade rate between 0.5/s and 3.0/s. The above algorithm was applied for the analysis period of -500 to 500 ms of stimulus onset. We did not find any significant difference in the microsaccade rate across all 25 contrast conditions for most of the stimuli in either monkey by performing 1-way ANOVA test on microsaccade-rate (Monkey 1: P_static_ = 0.19, P_counterphase_ = 0.45, Monkey 2: P_static_ = 0.12, P_counterphase_ = 0.02). Therefore, the results shown here cannot be attributed due to potential differences in eye positions or movements across stimulus conditions.

## Discussion

We used plaid stimuli composed of 2 superimposed orthogonal gratings with varying contrasts to alter normalization strength and determined how that affects different neural measures such as multiunit spiking activity, gamma power, high-gamma power, and SSVEP power in V1. Although normalization affected these measures in different ways, a single normalization model, adapted for population responses, provided excellent fits to all the data.

### Previous Studies on the Effect of Normalization on Different Neural Measures

#### Spiking Activity

Cross-orientation suppression has been best studied in spiking activity, both extracellular (DeAngelis et al. 1992; Carandini et al. 1997; Freeman et al. 2002) and intracellular (Bishop et al. 1973; Morrone et al. 1982; Priebe and Ferster 2006). Our N.I. values are closer to those observed by Ruff and Cohen (Ruff et al. 2016) who also recorded using the chronically implanted Utah arrays (1.14, 1.04, and 1.08 for multiunits recorded from V1, MT, and V4). In our recordings, the spatial frequency was further restricted to 4 cpd to maximize gamma power, which may not have been optimal for spiking activity resulting in slightly smaller values of N.I. compared with Ruff and colleagues in V1. However, the observed weak cross-orientation suppression in the small receptive fields of V1 can also be explained by the decrease in the surround suppression provided by plaid stimuli, as shown by Walker et al. (2002).

#### Gamma Power

Our results were consistent with Lima et al. (2010) who also showed strong suppression of gamma for plaid stimuli in V1. However, they did not study multiple contrast levels for both gratings; neither did they fit any model to their data. Lima and colleagues interpreted their results by stating that single gratings induce strong cooperative interactions between population of neurons, whereas plaid stimuli generate competition between different population of neurons. Suppressed gamma responses for plaid stimuli have also been shown in ECoG recordings by Hermes et al. (2019).

Although our results were well explained using the modified normalization model, it is unlikely that populations of neurons that prefer orthogonal orientations exert a >3-fold suppressive effect (median N.I. 3.37, range 1.8–6) on each other. Instead, gamma oscillations are likely to arise due to excitation–inhibition interactions (Whittington et al. 1995; Bartos et al. 2007; Cardin et al. 2009; Sohal et al. 2009; Buzsáki and Wang 2012; Veit et al. 2017), which have been modeled using very different methods (Wang 2010), such as Wilson–Cowan type models of excitatory– inhibitory balance (Wilson and Cowan 1972; Jia et al. 2013; Jadi and Sejnowski 2014), “interneuronal network gamma” models (Whittington et al. 1995; Wang and Buzsáki 1996), and “pyramidal inter-neuronal network gamma” models (Whittington et al. 2000; Tiesinga and Sejnowski 2009). Therefore, gamma power itself may not be a good measure of normalization because it may not necessarily rely only on normalization mechanisms. Recently, the reduction in gamma power due to the presentation of plaid stimuli has been explained by a variance-based model (Hermes et al. 2019), in which the gamma responses are largely driven by the variance across orientations in the population receptive field. Plaid stimuli activated multiple orientation columns and reduced the variance, and therefore reduced gamma power. We have previously shown that gamma power varies with normalization strength, but the relationship is nontrivial, possibly due to the complex excitation–inhibition interactions that underly normalization (Ray et al. 2013). Although these studies suggest that it may be too simplistic to model gamma power using a normalization model as done here, it is nonetheless an important characterization because gamma power has also been reported to be coupled to other neural measures such as functional magnetic resonance imaging (fMRI) (Lachaux et al. 2007; Nir et al. 2007), which have been routinely used to study CRFs and their modulation with behavioral state such as attention (Buracas and Boynton 2007; Li et al. 2008). Therefore, to interpret these results in the normalization framework, it is crucial to study its effect on other neural measures related to the blood oxygen leveldependent signal, such as gamma oscillations.

#### High Gamma

High-gamma power in the LFP has been linked to firing rate of neurons around the microelectrode (Ray et al. 2008; Manning et al. 2009; Ray and Maunsell 2011; Ray 2015). To our knowledge, no study has looked at the effect of cross-orientation suppression in the high-gamma of the LFP. However, Hermes and colleagues have studied the effect of plaids on both gamma and high-gamma in human ECoG. They observed that the “Broadband power” (30-200 Hz), spanning both gamma (32-80 Hz) and high- gamma ranges (104-248 Hz) for our study, increases with the number of component gratings, whereas “narrowband gamma power” (30-80 Hz) decreases with the number of component gratings, in agreement with our observations. While gamma was related to the variance between orientation columns, high- gamma was related to the mean activity in these columns, similar to a model developed for fMRI (Kay et al. 2013).

#### SSVEP Power

Many EEG studies have studied interactions between multiple visual stimuli at different temporal frequencies and modeled these interactions using normalization mechanisms (Candy et al. 2001; Tsai et al. 2012). Tsai et al. (2012) used temporally modulated stimulus contrast in the numerator of the normalization equation and time-varying normalization pool in the denominator and used exponents to scale responses unlike our model to explain SSVEP responses as well as intermodulation terms. In our data as well, SSVEP responses were well explained by all the models, including the 3-parameter untuned normalization model (Fig. 7A).

We used static gratings to analyze spike and LFP responses for gamma, high-gamma power while using counterphase flickering gratings for SSVEP power. Counterphase stimuli were not used for all the measures because they generate strong SSVEP signals at second harmonic and all subsequent even harmonics. In our study, we used 8 Hz counterphase gratings causing the SSVEP signal to be found at 16,32,48 Hz, and so on, thus interfering with gamma and high-gamma responses. To avoid this, we used separate stimuli to measure and analyze the normalization trends for different neural measures. However, use of static and counterphase flickering stimuli to obtain different neural measures complicates the comparison of normalization measures across different types of neural responses.

### Comparison of Our Model With Other Normalization Models

In our study, we have compared predictions of 3 normalization models—untuned (equation 1), tuned (equation 2), and population (equation 3) and found that the population model outperforms others, especially for the gamma CRFs, even though the latter 2 have the same number of free parameters. The key difference is that only in the population model, the tuned normalization parameter (α) can be arbitrarily increased without decreasing the responses to the gratings-only conditions, in which either c1 or c2 is set to zero. Specifically, if we ignore the semisaturation term (σ) from the normalization equations, the responses to presentation of grating 1 (for which c_2_ = 0) and grating 2 (c_1_ = 0) are L_1_ and L_2_/α for the tuned normalization model but are L_1_ and L_2_ for the population model. Therefore, a condition in which the plaid condition produces a weaker response than the constituent gratings can be readily achieved in the population model by appropriate values of L1 and L2 and a large value of α, but this cannot be done for the tuned normalization model because increasing α decreases the response to grating 2 presented alone. We tried another version of the tuned normalization model in which *α* term was multiplied to c_1_, but this did not improve the overall fit quality because now increasing α reduced the responses to grating 1 alone. Similarly, a weighted sum model proposed by Busse et al. (2009) cannot explain the results for gamma oscillations because the population response cannot be lower than the response of both stimuli presented alone. Interestingly, our model is similar to the equal maximum suppression (EMS) spatially tuned normalization model or EMS-stimulus tuned normalization model (equations 7 and 8 of Ni and Maun- sell 2017), which was used to explain MT responses to both individual or multiple stimuli present at different locations inside the large receptive fields of MT area when attention was directed to different locations of the receptive field. They used this model to explain mutual suppression by populations of neurons encoding the preferred or null stimuli inside the receptive field. In our model, we used one normalization parameter (α) instead of using 2 normalization parameters used by Ni and Maunsell, because in our case there was no clear preferred or null stimulus and the model did very well with only a single tuned parameter.

Although our model was able to explain the populationlevel neural measures, it is descriptive in nature and does not provide a comprehensive biophysical explanation of why the normalization strength is different for different measures. It also lacks dynamics (fluctuations in normalization strength), which has been introduced in recent normalization models (Tsai et al. 2012; Zhou et al. 2019) and cannot explain differences in crossorientation normalization produced by overlapping gratings of arbitrary relative orientations, as discussed and implemented in other normalization models (Candy et al. 2001; Hermes et al. 2019). Our analysis was limited to variants of the standard normalization model, because these were directly comparable to a large body of literature that have used similar simplistic models to explain spike responses (Ni et al. 2012; Ruff et al. 2016; Ni and Maunsell 2017). Also, more complex models (Candy et al., 2001, Hermes et al. 2019, Zhou et al., 2019) were fitted over a larger set of features (such as multiple relative orientations and temporal frequencies) than our dataset. Future studies involving a larger set of stimulus parameters and more detailed modeling are needed to explain the biophysical origins of different forms of normalization reported here.

### Relation Between Normalization Strength and Attention on CRFs

Our results partially reconcile some results from attention studies that have shown different effect of attention on CRFs. Specifically, while some single-unit studies have shown that attention enhances neuronal spike response for moderate-contrast stimuli more than very low or very high contrasts, resulting in a leftward shift of CRFs (contrast gain; Reynolds et al. 2000; Martínez-Trujillo et al. 2002), others have shown a multiplicative boosting of neural responses at all contrast levels resulting in an upward shift of CRFs (response gain; Williford and Maunsell 2006; Lee and Maunsell 2010). Both contrast and response gain in attentional shifts have been also observed in studies involving psychophysics and behavior in humans (Ling and Carrasco 2006; Pestilli et al. 2009; Herrmann et al. 2011), LFP band powers in macaques (Chalk et al. 2010), and SSVEP power from EEG (Di Russo et al. 2001; Joon Kim et al. 2007; Lauritzen et al. 2010; Itthipuripat et al. 2014). The differences in single-unit studies have been largely reconciled based on a normalization model in which factors such as the size of the stimulus and the attention field can lead to contrast or response gain like changes in the CRF (Reynolds and Heeger 2009), and variants of such normalization models (including tuned normalization) have recently been used to explain the effect of attention on stimulus interactions as well as noise correlations (Ni et al. 2012, 2019; Ni and Maunsell 2017; Verhoef and Maunsell 2017). Although we did not manipulate attention directly in our study, we show that normalization itself has different effect on different neural measures, and therefore provides another factor that could lead to differences in the way CRF obtained from different neural measures shift due to attention.

## Supplementary Material

Supplementary material can be found at Cerebral Cortex Communications online

Supplementary Information
