# Supplementary material for: Effect of Cross-Orientation Normalization on Different Neural Measures in Macaque Primary Visual Cortex
Source: Cereb Cortex Commun. 2021 Feb 10;2(1):tgab009. doi: 10.1093/texcom/tgab009 (PMC8152940; doi:10.1093/texcom/tgab009)
Supplement: Supplementary file 1 [file supplementary_information_tgab009.docx]

**Supplementary Information**

**Supplementary Figure 1**

Population spiking activity profile for counterphase gratings. Same layout as Figure 4.

**Supplementary Figure 2**

Comparison of normalization model parameters for all neural measures. (A) Bar plots (white to gray) showing median explained variance for the respective neural measures for unique electrodes from Monkey 1 (N=37; left column) and Monkey 2 (N=13; right column). Individual electrode data (filled black circles) have been overlaid on top of bar plots. (B) Bars (white to gray) represent median corrected AIC values for standard, tuned and “population” normalization models for same set of electrodes from the two monkeys. (C) Bar plots of median normalization parameter (α) for all four neural measures for the population model for Monkey 1 (dark gray) and Monkey 2 (light gray). (D) Bar plots of median semi-saturation parameter (σ) for all four neural measures for the population model for Monkey 1 (dark gray) and Monkey 2 (light gray). Error bars indicate bootstrapped standard error of the median. 

**Supplementary Figure 3**

Comparison of normalization model parameters for all neural measures for non-unique electrodes. Layout same as in Supplementary Figure 2 but for N=143 non-unique electrodes in Monkey 1 and N=48 in Monkey 2. 

**Supplementary Figure 4**

Same as Figure 7, but when the analysis interval was set to the early stimulus period (0 to 250 ms, where 0 indicates stimulus onset).
